# FLUX: A pipeline for MEG analysis

**DOI:** 10.1016/j.neuroimage.2022.119047

**Published:** 2022-06

**Authors:** Oscar Ferrante, Ling Liu, Tamas Minarik, Urszula Gorska, Tara Ghafari, Huan Luo, Ole Jensen

**Affiliations:** a Centre for Human Brain Health, School of Psychology, University of Birmingham, UK; b School of Communication Science, Beijing Language and Culture University, Beijing, China; c Center of Functionally Integrative Neuroscience, Department of Clinical Medicine, Aarhus University, Aarhus, Denmark.; d Department of Psychiatry, University of Wisconsin–Madison, Madison, WI, USA; e Department of Physiology, Medical School, Shahid Beheshti University of Medical Sciences, Tehran, Iran; f School of Psychological and Cognitive Sciences, Peking University, Beijing, China

**Keywords:** Magnetoencephalography, Pre-registration, Replicability, Pre-processing, Event-related fields, Multivariate pattern analysis, Decoding, Source modelling

## Abstract

Magnetoencephalography (MEG) allows for quantifying modulations of human neuronal activity on a millisecond time scale while also making it possible to estimate the location of the underlying neuronal sources. The technique relies heavily on signal processing and source modelling. To this end, there are several open-source toolboxes developed by the community. While these toolboxes are powerful as they provide a wealth of options for analyses, the many options also pose a challenge for reproducible research as well as for researchers new to the field. The FLUX pipeline aims to make the analyses steps and setting explicit for standard analysis done in cognitive neuroscience. It focuses on quantifying and source localization of oscillatory brain activity, but it can also be used for event-related fields and multivariate pattern analysis. The pipeline is derived from the Cogitate consortium addressing a set of concrete cognitive neuroscience questions. Specifically, the pipeline including documented code is defined for MNE Python (a Python toolbox) and FieldTrip (a Matlab toolbox), and a data set on visuospatial attention is used to illustrate the steps. The scripts are provided as notebooks implemented in Jupyter Notebook and MATLAB Live Editor providing explanations, justifications and graphical outputs for the essential steps. Furthermore, we also provide suggestions for text and parameter settings to be used in registrations and publications to improve replicability and facilitate pre-registrations. The FLUX can be used for education either in self-studies or guided workshops. We expect that the FLUX pipeline will strengthen the field of MEG by providing some standardization on the basic analysis steps and by aligning approaches across toolboxes. Furthermore, we also aim to support new researchers entering the field by providing education and training. The FLUX pipeline is not meant to be static; it will evolve with the development of the toolboxes and with new insights. Furthermore, with the anticipated increase in MEG systems based on the Optically Pumped Magnetometers, the pipeline will also evolve to embrace these developments.

## Highlights

• We propose a pipeline for MEG research making analysis steps and parameters explicit.
• The FLUX pipeline is developed to be used with MNE Python and FieldTrip and it includes the associated documented code.
• The pipeline includes the state-of-the-art suggestions for noise cancellation as well as source modelling including pre-whitening and handling of rank-deficient data.
• To facilitate pre-registration and precise reporting we provide concrete suggestions on parameters and text to document.
• An example data set allows for the pipeline to be used in educational settings.

## 1 Introduction

Magnetoencephalography (MEG) has become an important tool in cognitive and clinical neuroscience (Baillet, 2017). Research involving MEG relies heavily on data analysis for quantifying the signals in the time and frequency domains, as well as for source modelling. MEG research has strongly benefited from open source toolboxes facilitating the analysis (Baillet, 2017; Gramfort, 2013; Hinkley et al., 2020; Litvak et al., 2011; Oostenveld et al., 2011); however, many analysis options both within and across toolboxes present several challenges. As such, there is not a common consensus on how best to perform basic steps such as artifact rejection or source modelling and in which order the steps of these analyses should be performed. Even within a given toolbox, several approaches can be taken to perform a specific analysis step and there are multiple parameters to be adjusted. While the many degrees of freedom allow for optimizing the analysis in the context of a given paradigm, it may be at the expense of a ‘best-practice approach’ and it complicates matters for new researchers entering the field. This paper aims to define an analysis pipeline – termed FLUX – which is an attempt to work towards a best practice. The pipeline is compatible with more general guidelines for MEG research (e.g., Hari et al., 2018). The pipeline has been implemented using two toolboxes namely MNE-Python (Gramfort, 2013) and FieldTrip (Oostenveld et al., 2011); however, the steps and recommendations do apply to any other toolbox.

One key motivation for the proposed pipeline is to facilitate open science with the larger aim of improving the replicability of MEG research. If different approaches are used to address similar questions, this might hamper reproducibility. Also, when research is published, there is a great variation in details provided in the method section. Relying on a standard pipeline will make it simpler to describe the methodology and therefore facilitate future research building on the published findings. Pre-registrations are becoming increasingly important for empirical research (Shrout and Rodgers, 2018); however, they can be quite cumbersome due to the necessity of defining the analysis details before the study. The proposed FLUX pipeline will help this process and lower the threshold when researchers decide to pre-register a given study. The FLUX pipeline will also provide concrete parameter settings and text suggestions to be included in the Method sections of publication and pre-registrations (see Supplementary Material). Finally, we also provide a standard operation procedure (SOP) for the recording of MEG data. This will further facilitate pre-registrations as well as promote standardization across laboratories.

Over the years several guidelines and recommendations for MEG research have been published. The FLUX pipeline we are proposing is well aligned with the suggestions on pre-preprocessing and basic analyses as outlined in various papers (Andersen, 2018; Bigdely-Shamlo et al., 2015; Meunier et al., 2020Meunier et al., 2020; Niso et al., 2019)) as well as the guidelines described to ensure reproducible MEG research (Gross et al., 2013; Hari et al., 2018; Pernet et al., 2020). In terms of source modelling our approach is consistent with the guidance for forward modelling and beamforming described in recent publications (Jaiswal et al., 2020; Jas et al., 2018; Westner et al., 2021). What we provide in addition to these papers is a specific pipeline with documented code taking the raw data to sources analysis. We also provide concrete suggestions for what to report in publications and registered reports. The FLUX pipeline is implemented as specific code with documented tutorials and an example data set (https://neuosc.com/flux/). We find it important to define a full pipeline including as the pre-processing strategy will impact the details of the subsequent analyses. For instance, the rank deduction resulting from signal-space suppression impacts the parameters used in the source modelling.

There is a strong trend towards acquiring large datasets in cognitive and clinical neuroscience collected over multiple sites (Niso et al., 2016; Poldrack and Gorgolewski, 2014). The FLUX pipeline is developed as part of the Cogitate Consortium and it is based on an adversarial collaboration investigation conscious by collecting a large data set (Melloni et al., 2021). The consortium is developed in the context of open science with the aim of acquiring high-quality data using state-of-the-art analyses approach. The FLUX pipeline is derived from the best practice developed in the Cogitate Consortium. As learned from the consortium, efforts on big data as well as cross-group collaborations will benefit from the FLUX pipeline as it will provide a standard analysis approach that can be used across sites. In the FLUX pipeline we will make explicit which analysis steps can be done automatically and which require visual inspection and interactions of the data. These steps will facilitate the development of a fully automated pipeline benefitting the analysis of large datasets.

Finally, education is an important motivation for the FLUX pipeline. The research field of MEG is constantly growing as more systems are being installed around the world. Moreover, the advent of OPM-MEG systems (Boto et al., 2018; Hill et al., 2019) is thought to be less expensive and more versatile catalysing an even faster expansion on the number of MEG laboratories. In parallel, there is a strong push to share already recorded MEG data (Niso et al., 2016) meaning that a wider group of researchers will be able to analyse existing datasets. In sum, these developments will result in an influx of researchers that need to be trained in MEG analysis. We have therefore made the FLUX pipeline available to the community via a GitHub repository (https://github.com/Neuronal-Oscillations/FLUX/) organized in an open access website (https://neuosc.com/flux/) together with an example dataset. To facilitate the integration with education, the FLUX pipeline is implemented and shared through Jupyter notebooks for MNE-Python and Matlab Live Editor for the FieldTrip toolbox.

## 2 Approach

### 2.1 The FLUX pipeline provided via Jupyter notebooks and Matlab Live Editor

The FLUX pipelines have been developed using MNE-Python (Gramfort, 2013) and FieldTrip (Oostenveld et al., 2011) which are open-source toolboxes implemented in Python and Matlab, respectively. Both toolboxes are widely used by the MEG community. There are other excellent toolboxes such as BrainStorm (Tadel et al., 2011), SPM (Litvak et al., 2011) and NUTMEG (Hinkley et al., 2020) and the FLUX pipeline could be expanded in the future to include those as well. The FLUX pipelines can be accessed via a website (https://neuosc.com/flux/) and the scripts themselves are maintained on a GitHub repository (https://github.com/Neuronal-Oscillations/FLUX/). While the pipeline will evolve with time, the aim is to keep it fairly constrained and have basic functionality relatively static albeit subject to refinements. With time, new sections will be included to add functionalities. The pipeline for MNE Python is defined using Jupyter notebooks as this allows for easy integration of the code in combination with descriptions, as well as textual and graphical outputs. The FieldTrip pipeline is defined using the Matlab Live Editor for the same reasons. The code from the notebooks can be copied and integrated into standard scripts.

### 2.2 The dataset

**Fig. 1:**
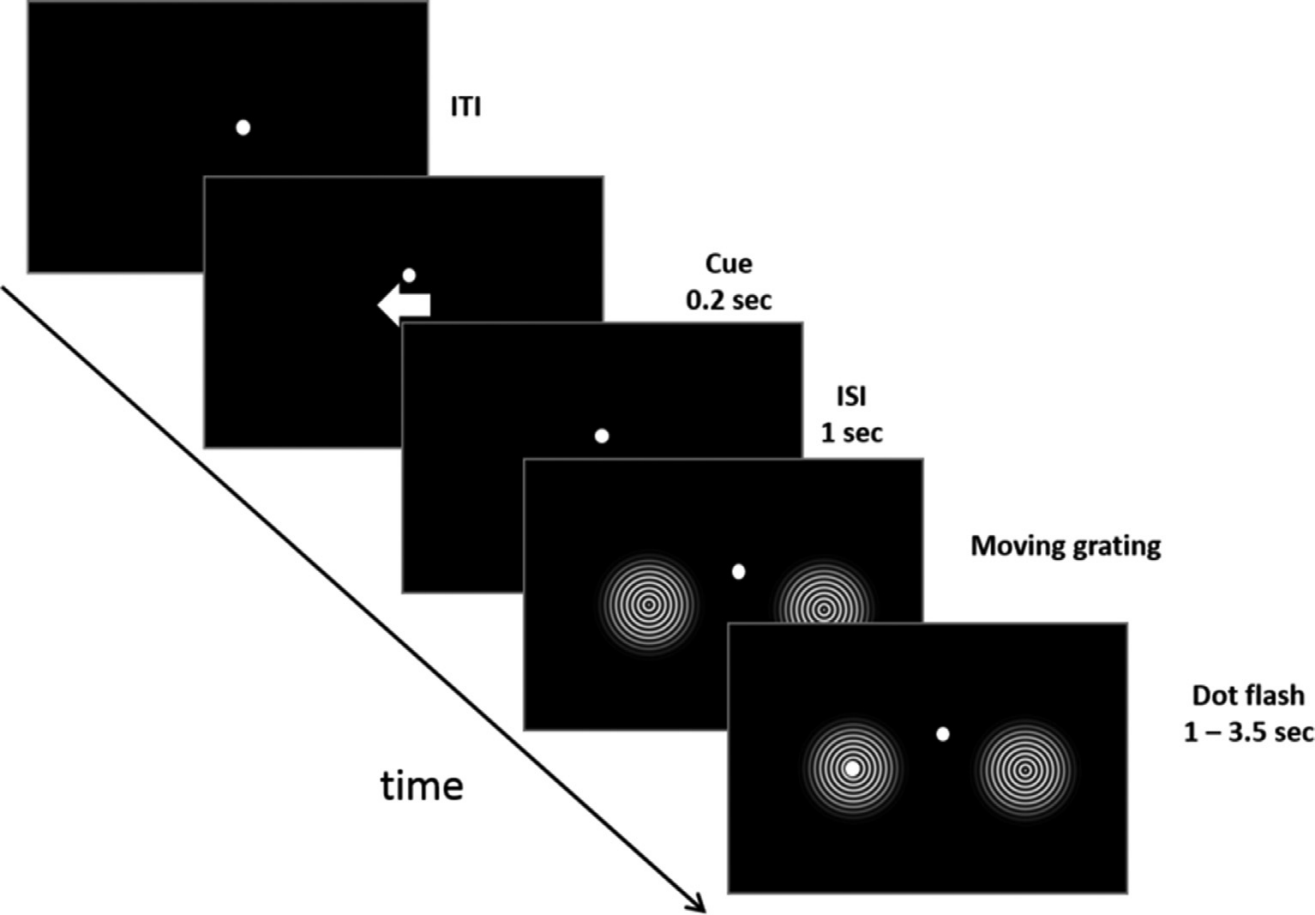
The paradigm used for the MEG data collection in the FLUX pipeline. A cue directs the participants to attend left or right. This results in strong hemispheric lateralization of the posterior alpha power (8–12 Hz). Then follows the presentation of the moving gratings which will induce strong gamma power (60–90 Hz) in the visual cortex as well as visual evoked fields. Finally, the button response to the *dot flash* will result in gamma band activity in the motor cortex followed by suppression in the beta band (15–40 Hz). Since the task produces robust and well-documented modulations in the alpha, beta and gamma band as well as event-related fields it is well suited for the FLUX pipeline with a focus on the characterization of oscillatory brain activity and source modelling.

To demonstrate the functionality of the pipeline we will be using an MEG dataset based on a spatial attention paradigm (Fig. 1). The paradigm is derived from a study involving moving gratings and the detection of subtle dots (based on; see also Hoogenboom et al., 2006). The dataset was recorded using the MEGIN Triux system at the MEG facility of the Centre for Human Brain Health, University of Birmingham, UK. Each trial in the paradigm started with a fixation dot followed by a cue pointing either to the left or right. This indicated to the participants which of the two upcoming stimuli to attend. After a 1 s inter-stimulus interval, two inward moving circular gratings were shown in both hemifields. After a random interval (1–3.5 s) a white dot was presented for 50 ms at the centre of the grating in the cued hemifield. Participants were instructed to press a button (right index finger) as soon as they detected the dot. The stimulus presentation was implemented in Matlab using the Psychophysics Toolbox (Brainard, 1997) and the code be found on the website. The main advantage of this task is that it produces robust modulations in the 8–12 Hz alpha band during the cue-grating interval. Furthermore, the moving gratings will induce strong activity in the 60–90 Hz gamma band. A single data set is used in the FLUX pipeline which is publicly available via https://www.neuosc.com . It will later be augmented to include more data sets for group analysis.

While the FLUX pipeline is developed for the analysis of MEG data, most of the steps can also be applied for the analysis of EEG data. Additional steps include re-references as well as different tools for detecting based sensors as described in detail in Bigdely-Shamlo et al. (2015). In terms of source modelling, a multi-shell forward model is required for EEG. If the intent is to combine EEG and MEG data for source modelling, pre-whitening is required in order to bring the data on the same scale. We will make this explicit below when relevant.

### 2.3 A standard operation procedure for data collection

The website includes a standard operation procedure (SOP) for MEG data acquisition. It is important to integrate the SOP with the data analysis pipeline. This will for instance allow for using a consistent naming convention for the EOG and ECG channels; this is important when identifying these channels in the analysis pipeline. The SOP also allows for making explicit the anatomical landmarks used for co-registering the MEG data with the structural MRI. Finally, some source modelling approaches will benefit from empty room data to be recorded in order to calculate the covariance matrix associated with the environmental noise.

#### 2.3.1 Ocular and cardiac artifacts

Is it recommended to acquire the electrocardiogram (ECG) as well as the horizontal and vertical electrooculogram (hEOG and vEOG). The ECG can be useful for procedures to automatically attenuate cardioballistic activity. The hEOG and vEOG can be used to identify saccadic and eye-blink artifacts, respectively. This information may seem redundant with the eye-tracker recordings; however, the EOG signal can be used to fully automatize the rejection of ocular artifacts during ICA. Moreover, this information could be important in case the eye-tracker fails or the signal quality is poor. It should also be mentioned that attaching ECG and EOG electrodes is relatively fast and uncomplicated and that most MEG devices already have a built-in system for them.

We also recommended using a fast eye-tracker (sampling at 300 Hz or more) to monitor eye positions to ensure that the participants maintain fixation as well as to track fast saccades (Juhola et al., 1985). To detect micro-saccades, which might confound neuronal activity in the gamma band (Yuval-Greenberg et al., 2008), binocular eye-tracking is recommended as it provides a better signal-to-noise ratio helping to detect small eye movements. It will also allow for measuring convergence with further analysis. It is convenient to directly sample the eye-tracking output together with the MEG data using auxiliary data collection channels on the acquisition system.

#### 2.3.2 Combining MEG and EEG?

Whether EEG is recorded together with the MEG data depends on the application. The advantage is that the EEG will provide complementary information to the MEG, as MEG for instance is blind to radial neuronal sources (Hämäläinen et al., 1993). Furthermore, there is a large established literature on event-related potentials (ERPs; e.g. the P300 and N400) in relation to cognition (Luck, 2014). To relate event-related fields (ERFs) recorded with MEG to the ERP literature, it is of great value to have the EEG signal recorded concurrently with the MEG. Moreover, MEG and EEG can provide convergent and complementary information in multivariate analyses (Cichy and Pantazis, 2017). The disadvantage of concurrent MEG and EEG recordings is that some participants’ heads will not fit in the MEG helmet after the EEG cap has been applied due to the height of the EEG electrodes. Also, the EEG electrodes will increase the distance between the MEG sensors and the scalp to a small degree, thus slightly reducing the signal-to-noise ratio. Finally, it costs considerable time to install the EEG cap (30–45 min for two trained researchers for a 64-sensor cap).

#### 2.3.3 Head position

In a typical MEG system, several coils are attached on the scalp to measure the head position. Their positions are digitized in 3D together with anatomical landmarks (including the periauricular points and the nasion) commonly using a Polhemus device. The location of these coils will be measured in the MEG system by passing high-frequency currents through them such that they generate localisable magnetic fields. This should be done at least before and after data acquisition as well as in the breaks between blocks. One can also perform continuous HPI measurements; however, this might introduce artifacts in the MEG data that need to be attenuated by a lowpass filter. We do not recommend this unless there is a strong need to measure head position continuously as might be the case in children or patients that move a lot. This will allow for the later application of algorithms to compensate for head movements.

#### 2.3.4 Active shielding and other artifacts

Some MEG systems are located in noisy environments, which require active shielding to attenuate external artifacts. It is recommended to only apply active shielding under these circumstances, as the approach could introduce unwanted artifacts.

To avoid artifacts from the clothing (e.g. zippers, buttons, bras and belts buckles) it is advisable to provide the participants with non-magnetic clothing (e.g. scrubs). If time permits, it is also recommended to record 5 min of resting-state data (eyes open) for each participant to assess the quality of the data as well as identify subject-specific artifacts (e.g. dental work and make-up).

#### 2.3.5 Empty room recordings

Before an experiment, we recommend performing empty room recordings (2–3 min). This allows for identifying unknown environmental artifacts as well as faulty or untuned sensors. It also allows for calculating the covariance matrix associated with the environmental noise which will benefit source modelling.

#### 2.3.6 Structural imaging

A T1 MRI should be recorded for each participant. This will allow for later source modelling mapping the neural activity onto the structural brain image of each participant, improving spatial resolution. For the sequence, we recommend MPRAGE or ideally FLASH but it is not essential as long as the T1 scan is of good quality (e.g. see https://surfer.nmr.mgh.harvard.edu/ for details). It should be mentioned that for realistic forward models to be used for EEG, the MRI requirements might be more demanding if one wants to construct a multi-shell model.

### 2.4 Data analysis

**Fig. 2:**
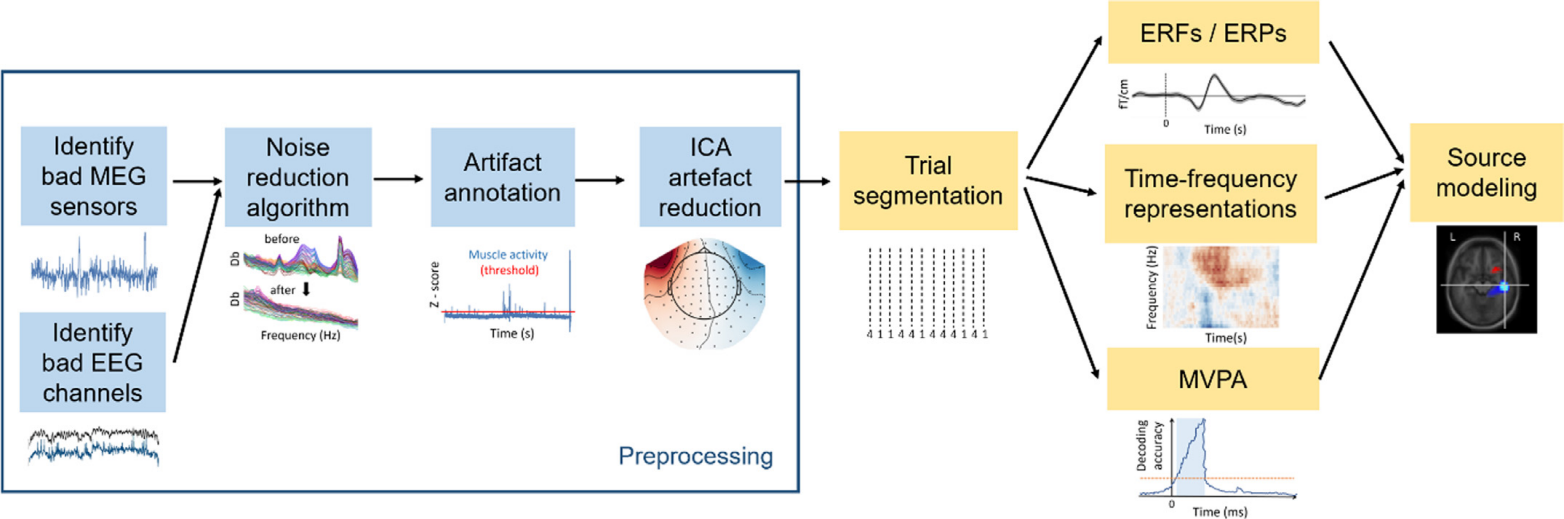
The flow-diagram representing the steps in the FLUX pipeline. First, the noisy sensors are marked for removal. MNE-Python then allows for annotating parts of the data with artifacts (e.g. due to saccades, muscle contraction, movements or sensor jumps). After, noise attenuations algorithms are applied (e.g. based on signal-space separation for MEGIN systems or the third-order gradient compensation for CTF systems). The ICA algorithm is applied for reducing ocular and cardiac artifacts. The data are segmented for trials specific for experimental conditions. At this stage trials with artifacts can be removed either based on the annotations or by semi-automatic threshold-based methods. Subsequently, the trial based data can be used to calculate event-related fields, time-frequency representation of power, or decoding using multivariate pattern analysis. Source modelling could then be performed to identify the sources responsible for producing the event-related fields or the modulations in power. These steps are detailed with documented scripts applied to a dataset in http://www.neuosc.com/flux/.

The flow of the data analysis is illustrated in Fig. 2 providing an overview of the pipeline. Below we describe the individual steps which are fully detailed on the FLUX website (https://www.neuosc.com/flux/).

#### 2.4.1 Pre-processing

Pre-processing of the MEG data is an important step as it serves to identify artifacts and attenuates various sources of interference. There are multiple paths for how to perform the pre-processing even within the same analysis toolboxes. Some of the options might be equally good, but we here settled on one approach.

#### 2.4.2 Noise reduction algorithms

Most MEG manufactures have algorithms for suppressing external noise sources that is applied in the pre-processing of the recorded data. For instance, the CTF system has a third-order gradient compensation algorithm making use of a reference array of gradiometers and magnetometers for noise reduction (Vrba and Robinson, 2001) whereas the MEGIN system employs a Signal-Space Separation (SSS; Taulu and Simola, 2006) approach which separates magnetic signals coming from within the sensor array from those coming from outside the array (i.e., environmental noise). These noise suppression algorithms all involve a linear transformation of the data. If one of the MEG sensors in the array is malfunctioning, this step would serve to spread artifacts from the faulty sensor to the rest of the sensors. Therefore, the first important step is to identify sensors with excessive noise as well as sensor producing signals with no signal variation (‘flat sensors’). After these malfunctioning sensors have been removed through interpolation, noise reduction algorithms can be employed. For the CTF system, it is recommended to use the third-order gradient compensation algorithm. For MEGIN system, we recommend using the Signal-Space Separation (SSS) and Maxwell filtering approach; however, keep in mind that these algorithms apply a regularisation step which reduces the rank of the data to 60–80 dimensions. This might cause problems later on, e.g. when performing the matrix inversion as done by some source modelling algorithms. As we will later address, this is best solved by calculated pseudo-inverse according to the reduced rank (Jas et al., 2018). For the MEGIN system, spatiotemporal SSS (tSSS) is also an option (Taulu and Simola, 2006). While this method can reduce head-movement artifacts, it does require continuous HPI measurements to operate optimally and several parameters must be adjusted. We recommend relying on SSS unless there are good arguments, such as excessive head movements, for using tSSS.

#### 2.4.3 Annotation of artifacts

The next step is to annotate artifacts in the MEG data to later remove problematic trials. This annotation is typically done by algorithms that can identify ocular artifacts and muscle contractions. It is always recommended to visually verify these annotations using a data browser. It should be mentioned that this annotation can be done in MNE-Python, whereas in FieldTrip artifacts typically are removed after trial segmentation. The strongest artifacts typically stem from muscle contraction, body movements, eye-blinks, saccades and malfunctioning MEG sensors. Muscle artifacts can be identified as high-frequency broadband signals (typically ranging from 110 to 140 Hz) particularly in the sensor around the rim. Saccades can be identified in the hEOG or from the eye-tracker and result in strong signals in frontal MEG sensors. Eye-blinks can be identified in the vEOG or the eye-tracker and likewise produce a strong frontal signal. Movement artifacts are observed as relatively slow signals which are strongly correlated in a large set of sensors. MEG sensor artifacts are typically observed as jumps in the ongoing signals and will be more frequent in some sensors than others. The section ‘artifact Annotations’ on the FLUX website (http://www.neuosc.com/flux) provides concrete examples of some of these artifacts and how they appear in the data. We recommend filtering the EOG at 1–10 Hz and the MEG at 110–140 prior to identifying respectively the ocular and muscle artifacts. Key parameters are the thresholds for what to consider ocular and muscle artifacts quantified by z-scores. It is recommended to first annotate the artifacts and then later consider whether to reject trials with artifacts depending on the research question. For instance, muscle artifacts are problematic when quantifying neuronal gamma-band activity due to the spectral overlap; however, these artifacts might be less problematic for later event-related fields (e.g., the N400m). MNE-Python offers a well-developed approach for artifact annotation. When using FieldTrip, annotations are typically not done first as artifact rejection is implemented after trial segmentation.

#### 2.4.4 Independent component analysis applied to reduce cardiac and ocular artifacts

The contribution of ocular and cardiac artifacts can be reduced using independent component analysis (ICA) (Vigario et al., 2000). To reduce the computation time, we recommend to down-sample a separate copy of the data to 200 Hz and applying a 1–40 Hz band-pass filter before applying the ICA algorithm. In MNE Python we have employed the FastICA method (Hyvärinen and Oja, 2000) and in FieldTrip the Infomax algorithm (Bell and Sejnowski, 1995). Possibly when applying ICA to EEG data there might be a benefit to include data at higher frequency ranges to e.g. identify muscle artifact (this is less relevant for MEG as the muscle artifacts are less spatially stable). When possible, it is advisable to reject segments with major movement and sensors artifacts before running the ICA algorithm. This can be done by telling the ICA algorithm to ignore segments with annotated artifacts or by rejecting the corresponding epochs. Subsequently, the ICA components reflecting eye-blinks, saccades and cardiac artifact are identified in the respective topographies and time-course by visual inspection. These artifacts typically appear in 3–5 components, but in some participants, they may not be easy to detect. Although it is possible to detect these components automatically (e.g., correlating their time course with the EOG/ECG signal), we here recommend identifying the artifacts manually until we better have assessed the automatic approaches. We do not recommend rejecting more components than a handful unless they are associated with a known artifact. After the artifactual ICA components have been identified, projections are made to attenuate the contributions of the respective artifacts. Note that these projections will be applied to the raw data which are not downsampled or filtered. When should one reduce ocular artifacts by ICA as compared to rejecting trials with artifacts? This will depend on the experimental question. For instance, if a study is conducted on covert spatial attention, it might be essential that participants keep fixation at the centre of the screen. In this case it is advisable to reject trials with saccades. However, in a study on auditory perception, one might choose to attenuate saccadic artifacts by ICA.

#### 2.4.5 Epoching the data according to condition-specific events

The next essential step is to segment the data according to the condition-specific events. This is typically done based on the information in the trigger channel defining the trial conditions. Often the behavioural output, e.g. button responses, are also taken into account. Depending on the research question, a pre- and post-stimulus interval must be defined. When segmenting the data, the artifact annotation can be applied to rejected trials in MNE-Python. When using FieldTrip, a semi-automatic approach can be applied to reject segmented trials with artifacts.

#### 2.4.6 Event-related fields

When calculating event-related fields (ERFs) we recommend adapting – when possible – the guidelines for event-related potentials (Luck, 2014; Woodman, 2010). Typical settings for investigating cognitive event-related brain responses would involve a 30 Hz lowpass filter and a 200 ms baseline. The length of the trials will depend on the experimental question. The visualization of event-related fields depends on the type of sensors in the system. The CTF system has axial gradiometers and fields can be visualized directly; however, it might be advantageous to transform the fields to the *combined planar gradient* (see e.g Fournier et al., 2010.). The combined planar gradient is calculated as the root-mean-square of the spatial derivative estimated for two orthogonal planar directions (however, for power estimates the combined planar gradients is typically calculated by summing the estimated power of the two orthogonal gradiometers). The MEGIN system has both magnetometers and planar gradiometers. When visualizing event-related fields of the planar gradiometers, the combined planar gradient might also be applied. When calculating the combined planar gradient for event-related fields, we recommend subtracting the baseline just before performing the root-mean-square of the planar gradiometer signals. It should be noted that, unlike event-related fields, the magnitude of the *combined planar gradients* is somewhat biased by trial number. The main advantage of applying the combined planar gradient is that the magnitude of this planar gradient signal is typically the strongest directly above the neuronal source (Hämäläinen et al., 1993). While this might aid interpretation for complex field patterns, the disadvantage is that the combined planar gradient conceals the dipolar patterns thus removing information on e.g. source orientation.

#### 2.4.7 Time-frequency representations of power

**Fig. 3:**
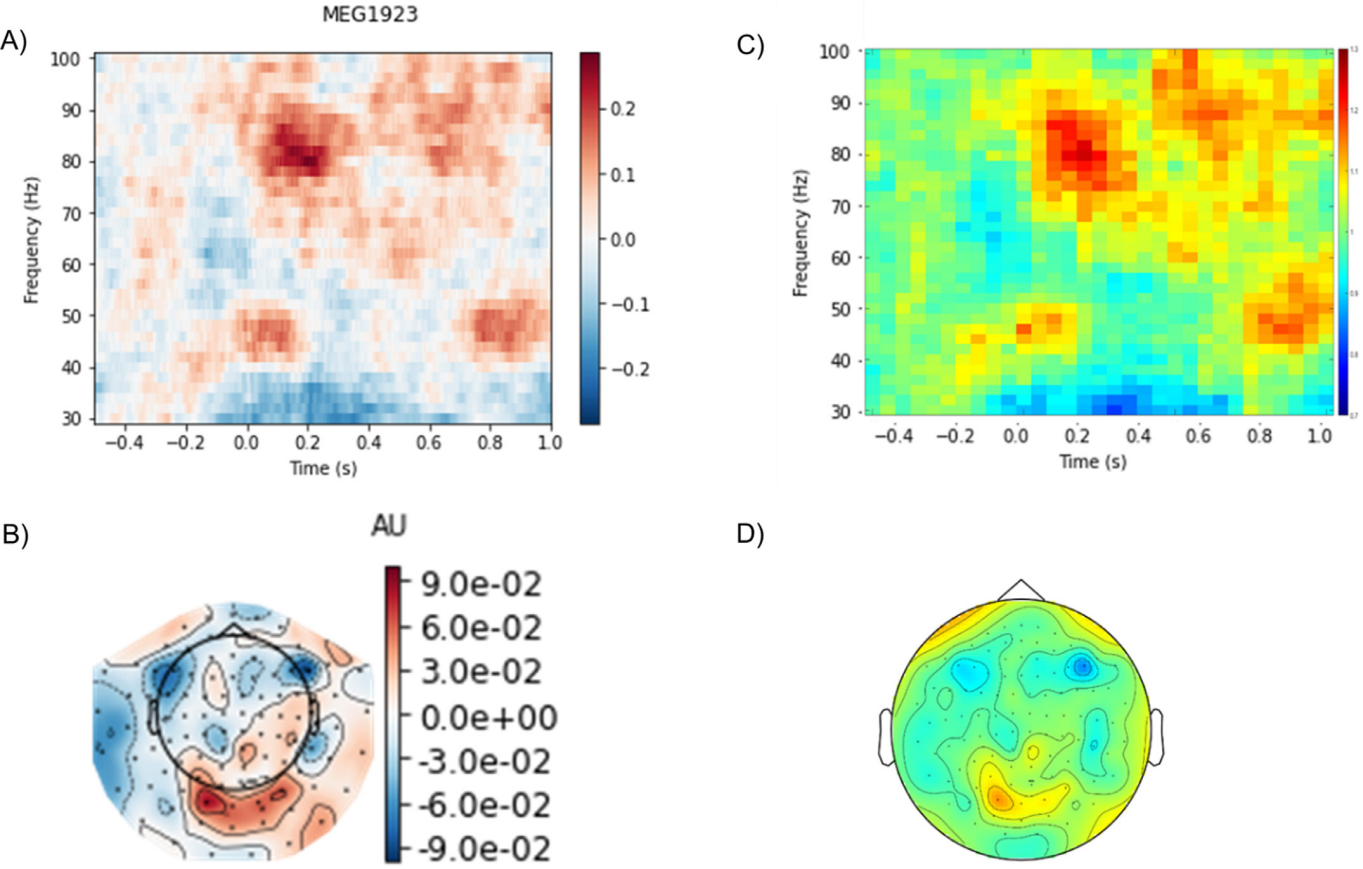
An example of time-frequency representations of power calculated for faster frequencies using the FLUX pipe They were calculated using MNE Python (A) and FieldTrip (B) using similar settings and yielded very comparable results. Note the relative increase in gamma power (80–90 Hz) in response to the onset of the gratings (*t* = 0 s). Also, the corresponding topographies for gamma power in the 0–600 ms interval were similar (B and D). The power was estimated using multi-tapers to optimize spectral smoothing. In the FLUX pipeline, we provide concrete suggestions for the settings for optimizing quantifications of oscillatory power modulations for slow (<30 Hz) and fast (>30 Hz) frequencies.

Over the years, there has been a growing interest in neuronal oscillations and their mechanistic role in supporting cognitive functions (Buzsáki, 2006; Hari, 1997; Jensen et al., 2007). Typically, modulation of oscillatory brain activity is quantified using time-frequency representations of power. While wavelet approaches initially were used for this purpose (Tallon-Baudry, 1999), Fourier-based approaches including Hilbert transforms have become increasingly used. These methods are not fundamentally different and will produce similar results (Bruns, 2004; van Vugt et al., 2007). Nevertheless, it is essential to set the parameters correctly for these methods to optimize the spectral estimates. We here recommend a sliding time-window Fourier-based method for calculating time-frequency representations of power. Based on empirical observations, brain oscillations at slower frequencies (4–30 Hz; i.e. theta, alpha and beta band) are relatively narrow-band whereas they are more broadband in the gamma range (defined at 30–100 Hz). To accommodate these differences, we propose different settings for quantifying slow and fast brain band oscillations in the frequency domain. For slow activity (<30 Hz), we advise using a Δ*T* = 500 ms sliding time window. A single taper (also 500 ms) must be applied prior to the Fourier transformation preceding the power estimate. In FieldTrip, a 500 ms Hanning taper can be used while in MNE-Python a single taper from the Slepian sequence is recommended. These will result in about ∼3 Hz spectral smoothing. For the faster frequency range (>30 Hz) we recommend a multitaper approach (Percival and Walden, 1993) with a 250 ms sliding time window and *K* = 5 Slepian tapers. This will result in ∼10 Hz spectral smoothing (as *K* < 2 ΔTΔF). Time-frequency representations of power can be inspected either by performing a baseline subtraction or by comparing conditions. For Δ*T* = 500 ms, the recommended baseline is −750 to −250 ms as it does not include the post-stimulus interval when considering the temporal smoothing from the sliding time window. The baseline time window may vary depending on the experimental design though. Generally speaking, this time window should not overlap with stimulus presentation or response-related events. Typically spectral power is shown as relative change with respect to the baseline, P_relative_ = (P_stim_ – P_baseline_)/P_baseline_ (Pfurtscheller and Lopes da Silva, 1999) and when comparing the difference between conditions, e.g. condition A and B: P_relative_ = (P_A_ – P_B_)/(P_A_ + P_B_). The relative change in power provides an intuitive metric that is all well-normalized across participants. However, there are also other options such as log-ratio (P_log-ratio_ = log (P_B_/P_A_)) and measures normalized by standard deviation such t-scores and z-scores. Those should be used with great care as the distribution of power estimates is highly skewed resulting in large effect sizes being associated with a large standard deviation Fig. 3. shows an example of the relative modulation of power in the gamma band and the corresponding sensor level topography. Note that the power estimate for individual time-point points are a consequence of estimating the power using sliding time-window (being 250 ms for power estimates above 30 Hz and 500 ms below 30 Hz). This temporal smoothing must be considered when interpreting the power modulations.

#### 2.4.8 Source modelling

The topic of source modelling is complicated by the many approaches both in terms of forward as well as inverse modelling (Baillet et al., 2001; Hämäläinen et al., 2020). While source modelling in the past was done by dipole modelling estimating the location of a few discrete sources, various kinds of distributed source estimation have become state-of-the-art. We will here focus on methodology suited for localizing the sources of oscillatory brain activity, namely the Dynamical Imaging of Coherent Sources (DICS) approach (Gross et al., 2001). In terms of forward modelling, a realistic subject-specific single-shell model based on spherical harmonics fitted to the brain surface from the structural MRI (Nolte, 2003) is typically used in FieldTrip. The structural MRI is aligned with the MEG data according to anatomical landmarks. A boundary element model (BEM) is used in MNE Python (Gramfort et al., 2010) and it is constructed from brain-surface reconstruction using FreeSurfer (Dale et al., 1999; Fischl, 2012). Since the source modelling, in this case, is done on MEG data, a single-shell model is sufficient. However, if EEG is applied, a three-layer model is typically required. Typically, the DICS approach is used to identify the modulations of activity in frequency ranges identified using time-frequency representations of power. Often one estimates the sources for a specific time window. We recommend using similar settings in terms of length of time windows and tapers as used in the time-frequency analysis. This DICS approach works by defining a grid in the full brain volume, which then is scanned. This spacing of the grid is dependant on the research question and the point-spread function (Liu et al., 2002) but typically 5 mm is reasonable. Source estimates using beamforming approaches (LCMV and DICS) will have an increase in noise bias towards the centre of the head (Gross et al., 2001; Van Veen et al., 1997). This bias is best ‘subtracted out’ by comparing conditions relatively. This is typically the relative difference between two conditions or the relative difference in post- versus pre-stimulus power. Since the DICS approach relies on a spatial filtering algorithm making use of the cross-spectral density, it is important to use the joint cross-spectral density matrix for the conditions being compared (‘common spatial filter’). Special care should be taken when applying beamforming approaches to data on which the SSS has been applied for noise reduction as this step massively reduces the rank of the data (down to ∼70 for the MEGIN 306-sensors system) (see Jaiswal et al., 2020; Westner et al., 2021). The solution is to calculate the truncated pseudo-inverse ($X^{†})\;$using singular value decomposition after first estimating the rank (*k*) of the data (X):

$$X=U\Sigma V^{T}$$

$$X^{†}=V\Sigma_{k}^{-1}U^{T}$$

**Fig. 4:**
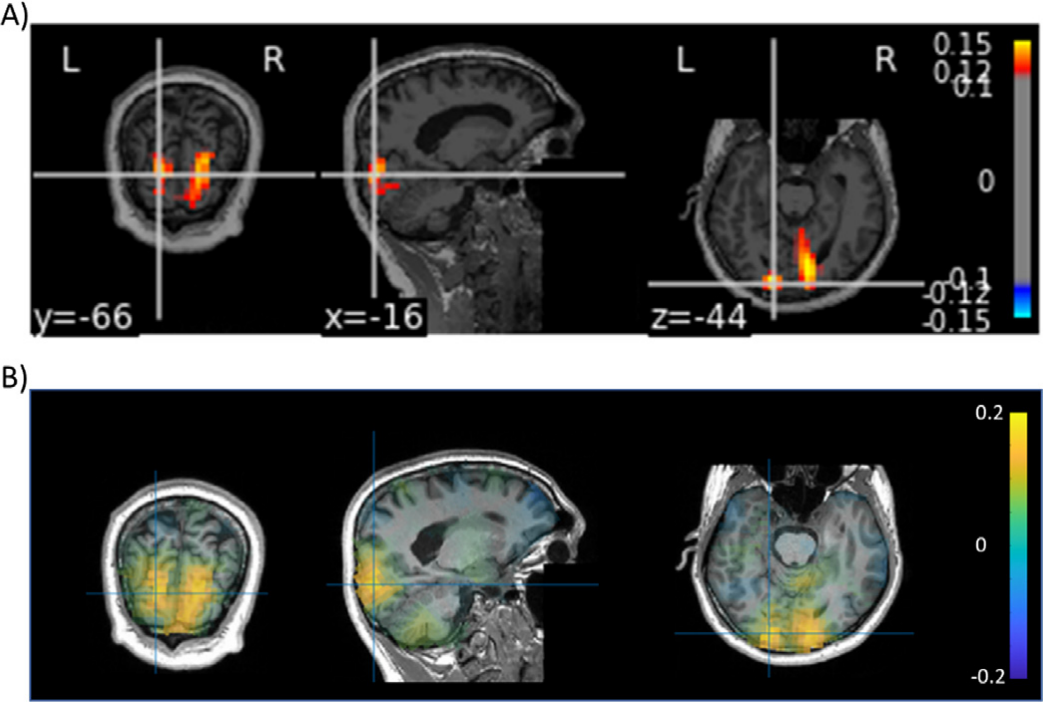
An example of source modelling of the gamma-band activity (60–90 Hz, 300–800 ms time window) was obtained using the DICS beamformer using MNE-Python (A) and FieldTrip (B). The results were very similar. We provide the scripts for obtaining the source modelling results including concrete suggestions for how to document the analysis path. In particular, we provide advice on how to reliably handle the rank reduction following the SSS. Note that for MNE Python both magnetometers and gradiometers after spatial prewhitening, whereas we FieldTrip we only used the gradiometers.

The truncation is implemented by considering the first *k* diagonal elements in Σ as well as the first *k* columns of U and V. The pseudo-inverse is then used in the beamforming algorithm. This approach efficiently handles the rank deficient data and – according to our experience - no further regularization is required. Finally, we also provide the settings for spatially pre-whitening the data. This can be done on either empty room data or data from the pre-stimulus interval. The pre-whitening aims to transform the empty room data to reduce the correlation between sensors (i.e. driving the off-diagonal elements in the covariance matrix towards zero). These steps also allow for bringing data from different sensors types on the same scale. In the FLUX pipeline, this allows for combining gradiometers and magnetometers for sources modelling, but it can also be used for combining MEG and EEG data. Subsequently, the source results are mapped onto the structural MRI of the participant. As shown in Fig. 4, the approach allows for localizing the increase in gamma power with the onset of the moving gratings.

In the future, we aim to expand the FLUX pipeline to include source estimates based on minimum-norm estimates as this approach also has proven very powerful in particular for event-related fields (Hauk et al., 2011; Lin et al., 2006).

#### 2.4.9 Multivariate pattern analysis (MVPA)

**Fig. 5:**
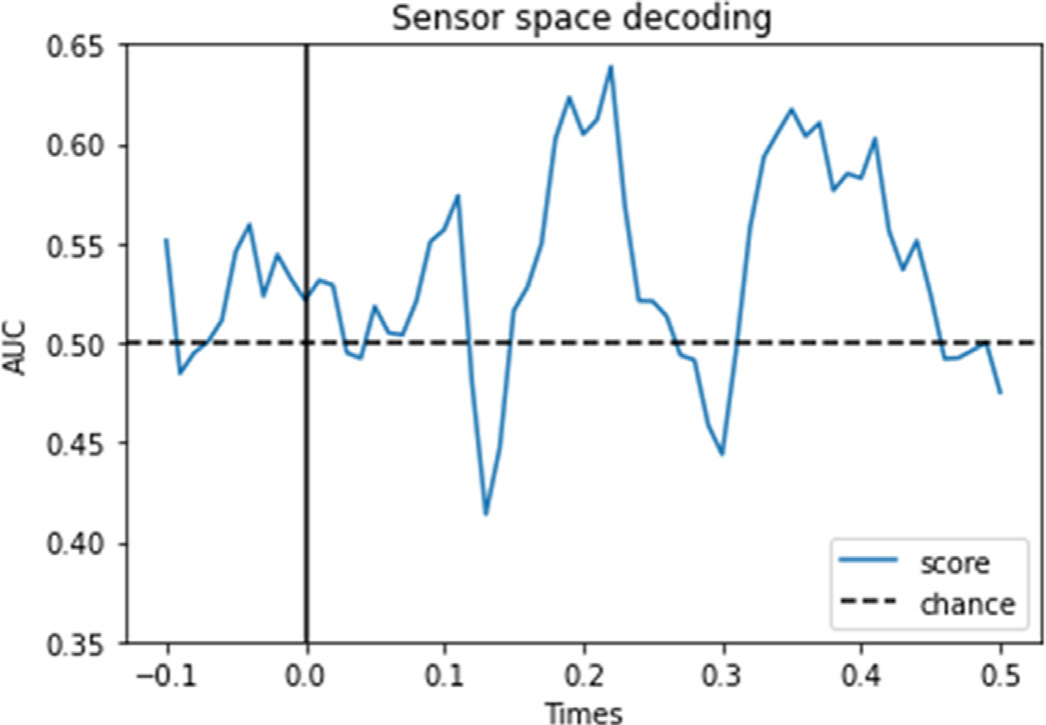
An example of multi-variate pattern analysis of the MEG data using the FLUX pipeline. In this example, trials associated with left versus right allocation of attention were decoded using a support vector machine (SVM). We applied MNE Python which uses the powerful Scikit-learn machine learning library. The outcome demonstrates that left versus right attention can be decoded ∼200 ms after stimulus onset.

Multivariate approaches are becoming increasingly important in the field of brain imaging including human electrophysiology (King and Dehaene, 2014; Stokes et al., 2015). The main idea is to identify representational specific patterns of brain activity by considering the activity distributed over the sensor or in source space. One widely used approach to MVPA is decoding using machine learning-based classifiers, in which different classes are predicted based on the distributed brain activity (Stokes et al., 2015; van de Nieuwenhuijzen et al., 2013). A second approach is representational similarity analysis (RSA) where similarity structures between stimuli or conditions are extracted from the neural signal and compared with the similarity structures of a specific representational model (Cichy et al., 2014; Cichy and Oliva, 2020; Guggenmos et al., 2018; King and Dehaene, 2014; Wang et al., 2018). One challenge of entering this research field is the many tools and types of classification approaches available. MNE-python offers many options in terms of MVPA approaches, while the choice is currently more limited in FieldTrip. In the example pipeline, we outline a simple approach based on classification utilizing support vector machines (SVM) (Cortes and Vapnik, 1995). We apply the classification approach to demonstrate that one can determine whether the participant is attending left or right based on the distributed brain activity. After the data are filtered (30 Hz lowpass) the classifier is trained using a leave-one-out approach. The example demonstrates that it is possible to obtain a reliable estimate of the attended direction (Fig. 5).

## 3 Discussion

We have here outlined the FLUX pipeline for the analysis of MEG data. The primary aim is to standardize analyses and parameter settings across toolboxes such as MNE-Python and FieldTrip to improve the reproducibility of MEG research. A second aim is to provide concrete recommendations for what to report in publications and pre-registered reports. This will serve to increase rigour when reporting as well as lower the threshold for pre-registering studies. Finally, the FLUX pipeline will also serve an important educational purpose. It is developed around a test dataset that can be used in self-studies and educational settings.

### 3.1 Comparison between toolboxes

Since the FLUX pipeline is implemented for both MNE-Python and FieldTrip, it is of relevance to comparing the two toolboxes. One of the main advantages of MNE-Python is that it is written in Python. Python has evolved into a widely-used programming language for novel data science approaches. Python modules are under constant development by a huge open source community ensuring the availability of state-of-the-art data science tools; in particular, the machine learning modules are exceptional. These developments ensure that MNE Python is future-proof and will allow for further integration with novel machine learning tools as multi-variate approaches are becoming increasingly important. Since Python is free, MNE-Python can be used with no financial constraints allowing academics with limited resources to contribute to MEG analysis. The MNE-Python toolbox is well funded and supported by an active and dynamic group of developers integrating the latest analysis tools of the field. In terms of functionality, MNE-Python was initially developed for data from the MEGIN system; this is convenient when for instance applying the maxfilter (SSS) tools which are integrated (Taulu and Simola, 2006). However, there are no apparent barriers to applying it to data from other devices such as the CTF MEG system. One strong feature of the MNE-Python pipeline is the artifact-annotation of the data, which allows for performing the artifact identification only once even if the trial length or other factors are changed later. In terms of source modelling, the minimum-norm estimate is well-integrated in the toolbox and it has been applied in numerous applications and is particularly suited for the analysis of event-related activity and decoding analysis. Finally, the MNE-Python toolbox is well integrated with various multivariate tools (Pedregosa et al., 2011). This is becoming increasingly important as multivariate classification and representational similarity analysis approaches are becoming increasingly popular (Guggenmos et al., 2018; King and Dehaene, 2014; Stokes et al., 2015). Given that Python is widely taught in schools and universities also provide an argument for adapting MNE-Python (Ozgur et al., 2021).

The FieldTrip toolbox was initially written for the CTF system. As such, some features associated with the MEGIN system are not implemented as for instance the SSS algorithm. Although, it is still possible to perform SSS as a preprocessing step using the Linux software provided by MEGIN. The FieldTrip toolbox has been developed with a focus on examining oscillatory brain activity. As such, the spectral beamforming tool such as DICS is well tested and has been used in numerous publications; however, the minimum norm estimates geared towards event-related fields are less developed. In terms of multivariate analysis, the Fieldtrip toolbox does offer some options based on MVPA-Light (Treder, 2020); however, the options are quite limited compared to those offered by MNE-Python. The FieldTrip toolbox is implemented in Matlab, which does limit the user community to researchers from institutions making Matlab available.

### 3.2 The future of the FLUX pipeline

We will keep evolving the FLUX pipeline, by improving and refining the already defined steps as well as the documentation. We will also add functionality, e.g., including a section on group-level analysis based on cluster randomization approaches and expanding on task-based connectivity analysis including measures on Granger causality (Chen et al., 2006; Michalareas et al., 2016) and cross-frequency coupling (Hyafil et al., 2015; Jiang et al., 2015). To promote open science and data sharing, we will implement the functionality to convert the MEG data to the BIDS format, a standard format for the organisation of neuroimaging data (Niso et al., 2018). This will be done when the *derivatives* (i.e., the data output conventions) of the MEG BIDS format are further developed. We are also considering complementing the tutorials (https://www.neuosc.com/flux) with JSON files defining the essential parameters for a given analysis. This would be important for setting up large analyses involving many participants. This can easily be done using the code from the FLUX pipeline. Another important challenge for the FLUX pipeline is the advent of MEG systems based on optically pumped magnetometer (OPM) sensors (Boto et al., 2018; Tierney et al., 2019), which might be using different data formats as well as varying sensor types and configurations. It would be important for the FLUX pipeline and the toolboxes to keep up with these developments to avoid fragmentation in the community.

## 4 Conclusion

We here put forward a pipeline for MEG data analysis with a specific focus on cognitive neuroscience applications. The main aim of the pipeline is to provide a guide for constraining the analysis path for a particular project given the many options provided by existing toolboxes. The pipeline will serve to improve the replicability of MEG research, as it aims to standardize the analysis steps. Furthermore, it will facilitate well-documented publications and lower the threshold for preregistration of future studies as we provide concrete suggestions for what to report. Finally, the pipeline can directly be used in educational settings and thus help to improve the standard in the research field of human electrophysiology.

## Data code statement

The data and code are available from https://neuosc.com/flux.

## CRediT authorship contribution statement

**Oscar Ferrante:** Writing – original draft, Formal analysis, Resources. **Ling Liu:** Formal analysis, Resources. **Tamas Minarik:** Formal analysis, Data curation. **Urszula Gorska:** Formal analysis. **Tara Ghafari:** Resources. **Huan Luo:** Writing – original draft. **Ole Jensen:** Writing – original draft, Formal analysis.

## Appendix Supplementary materials

Image, application 1
