# Supplementary material for: FLUX: A pipeline for MEG analysis
Source: Neuroimage. 2022 Jun;253:119047. doi: 10.1016/j.neuroimage.2022.119047 (PMC9127391; doi:10.1016/j.neuroimage.2022.119047)
Supplement: Supplementary file 1 [file mmc1.docx]

**Supplementary material**

Examples of the text to be used in publications and pre-registrations. The text is meant to provide examples of the details to be reported but should be adapted according to the specific analyses and toolbox used. As this advice will evolve, readers are encouraged to check for updates at <http://www.neuosc.com/flux>

Software

The data were analysed using the open-source toolbox MNE Python v0.24.0 (Gramfort et al., 2014) / FieldTrip v20211118 (Oostenveld et al., 2011) following the standards defined in the FLUX Pipeline (Ferrante et al, 2021).

Data acquisition

The ongoing MEG data were recorded using the TRIUX^TM^ system from MEGIN. This system has 102 magnetometers and 204 planer gradiometers. These are placed at 102 locations each having one magnetometer and a set of two orthogonal gradiometers. The horizontal and vertical EOG data as well as the ECG were acquired together with the MEG data. The data were sampled at 1000 Hz and stored for offline analysis. Prior to sampling, a lowpass filter at ~330 Hz was applied. To record the horizontal EOG, a pair of electrodes were attached approximately 2.5 cm away from the outer canthus of each eye. To record the vertical EOG, a pair of electrodes were placed above and below the right eye in line with the pupil. The ECG was recorded from a pair of electrodes placed on the left and right collarbone. Four head position indicator coils (HPIs) were placed behind the left and right ear as well as on the left and right forehead just below the hairline. The positions of the HPIs, the nasion, the left and right preauricular points, as well as the surface points of the scalp, were digitized using a Polhemus^TM^ device.

Artifact attenuation by MaxFilter

Sensors with excessive artefacts were marked from the data sets using a semi-automatic detection algorithm (about 8 per participant). Subsequently, the Signal-Space Separation (SSS) method (Taulu et al., 2004) was applied to reduce environmental artefacts. SSS decomposes the data using multipole moments based on spherical harmonics and removes the component of the magnetic field originating from outside the MEG helmet.

Artefact annotation

Artefacts were annotated in the raw data such that they later could be used to reject trials if relevant. Ocular artefacts were identified in the vertical EOG data (bandpass filtered at 1 - 10 Hz) according to segments exceeding a threshold defined according to (max(EOG) - min(EOG))/4. Ocular artefacts were considered 500 ms long centred around the time they exceeded the threshold. Muscle artefacts were identified in magnetometers data bandpass filtered at 110 - 140 Hz. The filtered data were z-scored and segments exceeding a z-score of 10 were annotated as muscle artefacts.

ICA for attenuating artifacts

The data were down sampled to 200Hz prior to Independent Component Analysis (ICA) and bandpass filtered at 1 – 40 Hz. Next the fastICA algorithm (Hyvärinen and Oja, 2000) was applied to the segmented data as implemented in MNE-Python. Clear components containing cardiac artifacts and eyeblinks (identified in time course and topographies of the ICA components) were removed in each subject (3 – 5 over subjects) in the raw unfiltered data.

Extracting condition-specific trials

The data were segmented into intervals of 4.5 s, ranging from 2.5 s prior to stimulus onset and 2 s after. To ensure that no artifacts were missed, trials in which the gradiometers values exceeded 5000 fT/cm or magnetometers exceeded 5000 fT were rejected as well as trials previously annotated with muscle artifacts.

Event-related fields

Prior to calculating the event-related fields, the data were lowpass filtered at 30 Hz (non-causal finite impulse response filter implemented using a Hamming window and a 441 sample filter length). After averaging, a 100 ms baseline was subtracted.

Time-frequency representations of power

The time-frequency representations of power were calculated using a sliding time window approach applied to the single trials (Tallon-Baudry and Bertrand, 1999). For frequencies lower than 30 Hz we used a 500 ms time window. A single discrete prolate spheroidal taper was multiplied to each time window prior to calculating the discrete Fourier transform. This resulted in ±3 Hz spectral smoothing. The power was then estimated as the squared modulus of the Fourier transform. This was done for 1 to 30 Hz in steps of 1 Hz. For frequencies above 30 Hz, a multi-taper method approach (Percival and Walden, 1993) was used involving a sliding time window of 0.25 s. To ensure sufficient spectral smoothing to detect the gamma activity, a set of tapers derived from discrete prolate spheroidal (DPSS), or Slepian sequences, were multiplied to the time-windowed data. We used 3 tapers resulting in a spectral smoothing of ~±8 Hz. Following a discrete Fourier transform the power estimate for the respective tapers were averaged. This was done for 30 to 100 Hz in steps of 2 Hz. The power estimates per trial were subsequently averaged. The relative change in power was then considered with respect to a baseline (-0.5 – -0.25 ms for f < 30 Hz and -0.5 – -0.125 ms for f > 30 Hz).

Constructing the forward model

Structural magnetic resonance images (MRIs) were acquired using a 3 Tesla Siemens MAGNETOM Prisma whole-body scanner (Siemens AG; TE = 2 ms, and TR = 2 s). The raw T1-weighted images were converted from DICOM to NIFTI. The coordinate system of the participants' individual MRI was aligned to the anatomic landmarks (nasion as well as the left and right preauricular points) and the scalp shapes digitized before the recordings. The alignment according to the MEG sensor array was done relative to four digitized head position indicator (HPI) coils. A single shell boundary elements model (BEM) was constructed based on the brain surface derived using FreeSurfer (Dale et al., 1999; Destrieux et al., 2010). This was then used to construct a volumetric forward model (5 mm grid) covering the full brain volume. The lead field matrix was then calculated according the head-position with respect to MEG sensor array.

Source modelling using DICS beamforming

The Dynamics Imaging of Coherent Sources (DICSs) approach (Gross et al., 2001) was applied to localize the modulations of oscillatory brain activity. To localize the modulation in the alpha band, the cross-spectral density (CSD) was calculated for the 8 to 12 Hz band using a multi-taper approach applying 2 Hz spectral smoothing. The CSD matrix was then calculated for the 300 - 800 ms stimulus interval in which the gratings were shown as well as the -800 – -300 ms pre-stimulus interval (using one DPSS taper). The combined CSD was used with the forward model to create a common spatial filter. This was done by first estimating the rank of the data (being reduced to ~70 due to the SSS and ICA approaches). The data were spatially pre-whitened using the covariance matrix from the baseline interval which allowed for combining the gradiometer and magnetometer data in the source modelling. The truncated pseudo-inverse was then calculated based on the estimated rank (Jas et al., 2018). For each source, the orientation was optimized to maximize the power of the output. The spatial filter was then applied to the CSDs to calculate the relative change in alpha power. As similar approach was used for the gamma band now considering the 60 - 90 Hz band and 8 Hz spectral smoothing (resulting in 3 DPSS tapers). The pre-stimulus time-interval was -0.6 to -0.125 s and the post-stimulus (Cortes and Vapnik, 1995)interval was 0.125 to 0.6 s. The source data were then mapped to the structural MRI.

Classification

A multi-variate pattern analysis was applied to the MEG data to classify whether the participants attended left or right. The data were lowpass filtered at 10 Hz and then downsampled to 100 Hz. The time-points in the interval -0.1 to 0.5 s around the target onset was considered. The data were standardized by removing the mean and scaling to unit variance per sensor. A support vector machine (Cortes and Vapnik, 1995) from Scikit-learn (<https://scikit-learn.org/>) was applied to classify the data as a function of time. A 5-fold cross-validation procedure was used and the classificaiton rate was reported as Area Under the Curve

**References**

Cortes, C., Vapnik, V., 1995. Support-vector networks. Mach Learn 20, 273–297. https://doi.org/10.1007/BF00994018

Dale, A.M., Fischl, B., Sereno, M.I., 1999. Cortical Surface-Based Analysis: I. Segmentation and Surface Reconstruction. NeuroImage 9, 179–194. https://doi.org/10.1006/nimg.1998.0395

Destrieux, C., Fischl, B., Dale, A., Halgren, E., 2010. Automatic parcellation of human cortical gyri and sulci using standard anatomical nomenclature. NeuroImage 53, 1–15. https://doi.org/10.1016/j.neuroimage.2010.06.010

Gramfort, A., Luessi, M., Larson, E., Engemann, D.A., Strohmeier, D., Brodbeck, C., Parkkonen, L., Hämäläinen, M.S., 2014. MNE software for processing MEG and EEG data. Neuroimage 86, 446–460. https://doi.org/10.1016/j.neuroimage.2013.10.027

Gross, J., Kujala, J., Hämäläinen, M., Timmermann, L., Schnitzler, A., Salmelin, R., 2001. Dynamic imaging of coherent sources: Studying neural interactions in the human brain. PNAS 98, 694–699. https://doi.org/10.1073/pnas.98.2.694

Hyvärinen, A., Oja, E., 2000. Independent component analysis: algorithms and applications. Neural Networks 13, 411–430. https://doi.org/10.1016/S0893-6080(00)00026-5

Jas, M., Larson, E., Engemann, D.A., Leppäkangas, J., Taulu, S., Hämäläinen, M., Gramfort, A., 2018. A Reproducible MEG/EEG Group Study With the MNE Software: Recommendations, Quality Assessments, and Good Practices. Frontiers in Neuroscience 12, 530. https://doi.org/10.3389/fnins.2018.00530

Oostenveld, R., Fries, P., Maris, E., Schoffelen, J.-M., 2011. FieldTrip: Open source software for advanced analysis of MEG, EEG, and invasive electrophysiological data. Comput Intell Neurosci 2011, 156869. https://doi.org/10.1155/2011/156869

Percival, D.B., Walden, A.T., 1993. Spectral Analysis for Physical Applications. Cambridge University Press, Cambridge. https://doi.org/10.1017/CBO9780511622762

Tallon-Baudry, C., Bertrand, O., 1999. Oscillatory gamma activity in humans and its role in object representation. Trends in Cognitive Sciences 3, 151–162. https://doi.org/10.1016/S1364-6613(99)01299-1

Taulu, S., Kajola, M., Simola, J., 2004. Suppression of interference and artifacts by the Signal Space Separation Method. Brain Topogr 16, 269–275. https://doi.org/10.1023/b:brat.0000032864.93890.f9
